# Software tools for cell culture-related 3D printed structures

**DOI:** 10.1371/journal.pone.0203203

**Published:** 2018-09-04

**Authors:** Marton Gulyas, Miklos Csiszer, Elod Mehes, Andras Czirok

**Affiliations:** 1 Department of Biological Physics, Eotvos University, Budapest, Hungary; 2 Department of Anatomy & Cell Biology, University of Kansas Medical Center, Kansas City, KS, United States of America; Consiglio Nazionale delle Ricerche, ITALY

## Abstract

Three-dimensional (3D) printing technology allowed fast and cheap prototype fabrication in numerous segments of industry and it also became an increasingly versatile experimental platform in life sciences. Yet, general purpose software tools to control printer hardware are often suboptimal for bioprinting applications. Here we report a package of open source software tools that we developed specifically to meet bioprinting requirements: Machine movements can be (i) precisely specified using high level programming languages, and (ii) easily distributed across a batch of tissue culture dishes. To demonstrate the utility of the reported technique, we present custom fabricated, biocompatible 3D-printed plastic structures that can control cell spreading area or medium volume, and exhibit excellent optical properties even at 50 ul sample volumes. We expect our software tools to be helpful not only to manufacture customized in vitro experimental chambers, but for applications involving printing cells and extracellular matrices as well.

## 1 Introduction

The recent spread of 3D printing technology allowed fast and cheap prototype fabrication in numerous segments of industry and it also became an increasingly versatile experimental platform in life sciences. Bioprinting now is efficient and accurate to build in vitro tissue models with the potential to provide pathologically relevant responses and thus model human disease mechanisms. Bioprinted structures increasingly yield phenotypic endpoints that are comparable with clinical studies and can provide a realistic prediction of clinical efficacy [1]. Several excellent papers review the various materials and bioprinting systems as well as their promise of clinical applications [2–6].

As a relevant example, a recent study describes bioprinting of three dimensional, cell-laden, vascularized tissues that exceed 1 cm in thickness [7]. These constructs could be perfused on a microfluidic chip for long time periods exceeding six weeks. Remarkably, the device can integrate up to three tissue types (parenchyma, stroma, and endothelium)—differentiated from human mesenchymal stem cells (hMSCs) in a customized extracellular matrix environment. In a similar effort, an artificial vascular network was reported by 3D printing of rigid sugar filament networks, followed by embedding in a fibrin hydrogel. After dissolving the sugar, the resulting tunels can be populated with endothelial cells and perfused with blood under high-pressure pulsatile flow [8]. Using a novel coaxial printing method a functional blood vessel can be fabricated, where the lumen is filled with a water-soluble material, while the bio-ink for the wall is a composite of ECM proteins and endothelial progenitor cells [9]. The structure of the extracellular environment can be also shaped by 3D printing technology. As an important example, follicle explants can be cultured in a suitable gelatin mesh structure, and can be used as a functional ovarian implant in surgically sterilized mice [10]. Such implanted, follicle-seeded scaffolds become highly vascularized and ovarian function was fully restored: pups are born through natural mating and thrive through maternal lactation.

Affordable 3D-printing also allows the development of specialized devices for in vitro cell technology. As an example, 3D printed inserts can be used to grow and stimulate neurons within geometrically constrained compartments [11]. By fabricating structures in culture dishes, one can control cell spreading, hence local cell density, or by restricting medium volume, decrease the amount of necessary reagents. 3D-printing technology also allows a simple in-lab fabrication of channels and reservoirs in cell culture dishes—on a cruder scale than litography-based microfluidic chambers, but without requiring specialized equipment and at a fraction of the cost [12, 13]. While 3D-printers, with small modifications, are capable bioengineering tools, these applications also require software tools that have distinct requirements from general-purpose slicer applications that convert a 3D solid object into a sequence of machine movements, conventionally encoded in g-code [14]. Most importantly, bioengineering applications like cell printing or fabricating cell-scale environments often require well-defined, specialized motion patterns and an ability to reproduce it in parallel targets, like an array of culture dishes. Prescribing each machine movement by manually editing the low-level g-code sequence is laborious, error-prone and time-consuming. For this reason we developed a software package which can represent machine movements or g-code elements by simple functions of a high level programming language such as python or C#. We also created a graphic user interface, PetriPrinter, which distributes the programmatically defined printer movements into several culture dishes organized in a grid pattern.

## 2 Materials and methods

### 2.1 Cell lines and culture conditions

P31 cells were a kind gift from Prof. K. Grankvist (University of Umea, Sweden). 3T3 cells were obtained from American Type Culture Collection (ATCC; CCL-92). Primary hippocampal culture was a kind gift of Katalin Schlett and Krisztian Tarnok (Eötvös University, Hungary), and was established according to the animal welfare permit PEI/001/1108-4/2013 and PEI/001/1109-4/2013, in agreement with European Union and Hungarian legislation.

Cells were grown at 37°C in a humidified, 5% CO_2_, 95% air atmosphere. For the cell lines, Dulbecco’s Modified Eagle Medium (DMEM, Lonza) containing L-glutamine was supplemented with 10% fetal bovine serum (Invitrogen) and penicillin-streptomycin-amphotericin B (Lonza). Primary cultures of embryonic hippocampal cells were prepared from CD1 mice on embryonic day 17/18 according to previously published protocol [15] using Neurobasal (NB) medium supplemented with B27 (Invitrogen) containing 5% fetal calf serum (FCS, Sigma), 0.5 *μ*M GlutaMAX (Invitrogen), 40 *μ*g/ml gentamicin (Hungaropharma, Budapest, Hungary), and 2.5 *μ*g/ml amphotericin B (Sigma).

### 2.2 Cytotoxicity screening

Cytotoxicity was evaluated by measuring the cellular protein content according to previously published protocol [16]. Cells were plated in the inner 40 wells of a 96-well plate. After 48 h exposure to various amounts of polylactic acid (PLA), cell monolayers were fixed with 10% trichloroacetic acid and stained for 15 min with sulforhodamine B (SRB). Cells were repeatedly washed with 1% (v/v) acetic acid to remove excess dye. The protein-bound dye was dissolved in 10 mM Tris and the optical density of the sample was determined at 570 nm using a microplate reader (EL800, BioTec Instruments, Winooski, VT, USA).

### 2.3 Live/dead assay

To characterize overall cell culture health, the Live/dead viability/cytotoxicity Kit (Invitrogen) was used according to manufacturer’s protocol. Briefly, live cells were labeled with 4 *μ*M calcein-AM in cell culture medium (DMEM supplemented with 10% fetal bovine serum) at 37°C for 30 minutes. Subsequently, dead cells were labeled with 8 *μ*M ethidium homodimer-1 in cell culture medium at 37°C for 30 minutes. Cells were fixed with 4% paraformaldehyde in phosphate buffered saline.

### 2.4 Microscopy imaging

All images were made on a Zeiss Axio Observer Z1 inverted microscope with 10x Plan-Neofluar or 5x EC Plan-Neofluar objective. The microscope was equipped with a Zeiss Axiocam MRM CCD camera.

### 2.5 3D printing into Petri dishes

A fused fabrication filament-based machine (Ultimaker Original) was used for printing in Petri dishes (for the detailed protocol see http://dx.doi.org/10.17504/protocols.io.rpfd5jn). We used polylactic acid (PLA) filaments manufactured by Verbatim (product number: 55283, color: gray, diameter: 2.85 mm). Printing temperature was 230 °C, nozzle diameter was 0.4 mm, printing speed was 300-500 mm/minute, extruding rate was 0.027-0.041. For printing, 35 mm tissue culture grade Petri dishes were used (Greiner, Cellstar TC cell culture dish, item no.: 627160).

### 2.6 Sterilization

3D printing in Petri dishes took place in open environment. After the printing process dishes were sterilized with 70% (v/v) ethanol for 10 min then dried for 2 h under a sterile hood.

### 2.7 Quantitative analysis of image contrast

To measure the quality of phase contrast images, we utilized the Fiji programming environment [17]. First, wells were selected by an ellipse ROI (region of interest) applied to a composite mosaic image, built from multiple adjacent field of views. To detect the local brightness changes, images were filtered through a Sobel edge detector. Cells were identified by a suitable threshold value applied to the Sobel-filtered image. The Sobel filter values were then averaged over pixels belonging to cells yielding a score for each image.

## 3 Results and discussion

### 3.1 Software tools

**Fig 1:**
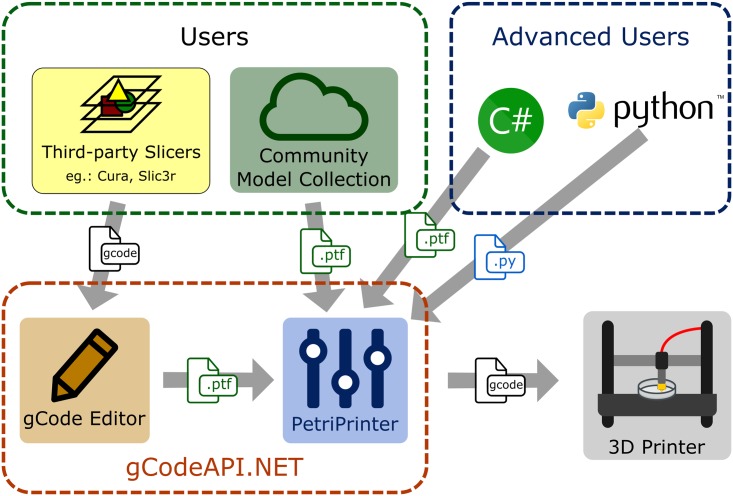
Overview of the various software tools and their applications. Geometrical objects can be encoded as python or C# files, as general use Standard Triangle Language (.stl) files, or as a sequence of machine specific commands (g-code). Internally, extrusion and movement commands are represented as a binarized gCodeCollection objects (.ptf) that can be edited and distributed into a number of parallel Petri dishes.

Our software consist of an application programming interface (API) and two graphic user interface applications (Fig 1). As described in more detail below, the API (gCodeAPI.NET) provides a convenient function library to represent g-code objects. The gCode Editor application visualizes g-code from third party slicer applications and allows their manual modification and optimization for cell culture applications. Finally, the PetriPrinter application distributes g-code objects into several culture dishes for batch printing. All parts of this software package require at least a Windows XP system and a Microsoft.NET Framework 4 package, and are licensed under the Apache License 2.0.

#### 3.1.1 gCodeAPI.NET

Native computer numerical control (CNC) programming language (g-code) is a very limited programming language which does not contain variables or logical operators and it is inconvenient to read by humans. This API encapsulates g-code commands into functions of high-level programming languages such as C# or python (see code examples in S1 Appendix). Besides the availability of the entire repertoire (variables, loops, flow control) of these languages, gCodeAPI.NET adds easily interpretable commands such as line, arc, relative and absolute position, speed, extrude or temperature. Documentation of the command library as well as code tutorials are available at the http://petriprinter.elte.hu website and on github (http://github.com/gulyasmarton/PetriPrinter). As demonstrated in the S1 Appendix, one can define a simple object like a cylinder surface with a few lines of code.

#### 3.1.2 gCode Editor

Generic purpose CAD programs allow the design of complex structures in a more convenient way than building it up from the elements of our g-code API. Slicer programs, which generate g-codes from a 3D solid model, do not offer a detailed control over the tool movements. When printing delicate structures, however, some extruder head movements may pull thin, undesired PLA threads and deposit them on the tissue culture surface. Close proximity of the hot extruder head and the tissue culture surface during head travel can also deteriorate the surface. Thus, there is a need to re-route such movements away from regions where cells are expected to adhere. Finding such problematic machine movements in the raw g-code, however, is quite difficult.

To make such tasks easier, our gCode Editor combines features of printing path viewers and text editors: One can navigate among the g-code commands while a parser function translates the code to human readable format and the Path Viewer draws the printing path. Using this visual information and GUI tools, one can identify and replace the problematic head movements: travel paths can be augmented by inserting new control points and existing control points can be relocated by mouse clicks. The output of the editor is an API object which is compatible with the PetriPrinter program.

#### 3.1.3 PetriPrinter

The PetriPrinter application is a g-code generator program which handles model files and aligns them into suitable positions to be printed into culture dishes. Using a graphic user interface one can assign an arbitrary combination of distinct objects to the available positions. The software provides collision-safe and optimized entry, exit and movement between the dishes.

Simple PLA-printable dish positioning objects like grids and rings are distributed as examples with the program package. The application can be adjusted to various hardware platforms by specifying settings such as printing temperature, start height, and row/column distances.

When using dish holders, determining the heights of culture surfaces is crucial to obtain reliable prints. According to our measurments, the average print-by-print variation of the culture dish surfaces is 0.2 mm (15 wells, 8 independent runs), larger than the height of a single layer. Thus, we augmented the printer with a microswitch probe to measure the surface height of each dish (see below), and utilize this information by the PetriPrinter software.

#### 3.1.4 Validation

To test if the gCodeAPI commands are translated into correct g-Codes, we translated test objects utilizing the basic elements of the API such as lines and ellipses. We extracted the coordinates from the generated g-Codes, and determined the distance between the points and the corresponding mathematical curve. For each object we found the distance between the g-Code points and the mathematical curve to fall wihin the numerical precision limit (10^−^8 mm).

### 3.2 Printing PLA objects in tissue culture dishes

#### 3.2.1 Modification of printer hardware

**Fig 2:**
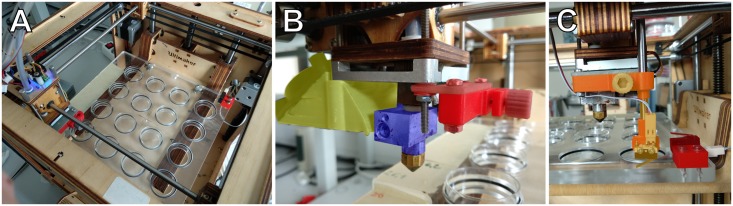
Adaptation of an ultimaker original printer for culture dish printing. A: Stage accepting an array of 35 mm cell culture dishes. B: Modified printhead. Yellow area: modified shroud to increase clearance to the stage and improve airflow. Blue area: a heater block that fits into the tissue culture dish. Red area: holder for a removable microswitch sensor, used to map the height of each dish. C: Printhead with microswitch sensor installed (orange). Another microswitch sensor, mounted on the stage (dark red) is used to calculate the height difference between the removable sensor and the nozzle.

To print into tissue culture dishes, we modified a hobby-level 3D printer (Ultimaker Original, Ultimaker B.V., The Netherlands). The fan shroud was changed to increase the rate of air flow and to allow an air gap between the top of the dish wall and the bottom of the shroud. The heater block was replaced by one with a cylindrical neck above the nozzle socket, in order to fit into culture dishes and to increase the printable area within the dishes (Fig 2).

To keep the tissue culture dishes at a pre-determined grid, one can either attach PLA-printed sockets to the build plate (object submitted to thingiverse upon acceptance), or replace the build plate with a dedicated stage where machined sockets are lined with O-rings to keep the dishes in place. In this latter approach the z coordinate of each dish is determined by a dedicated probe with a microswitch (Fig 2). This approach is precise (repeatable) within the resolution of the printer’s z axis positioning (12 *μ*m).

#### 3.2.2 PLA wells can provide optimal phase contrast optics

**Fig 3:**
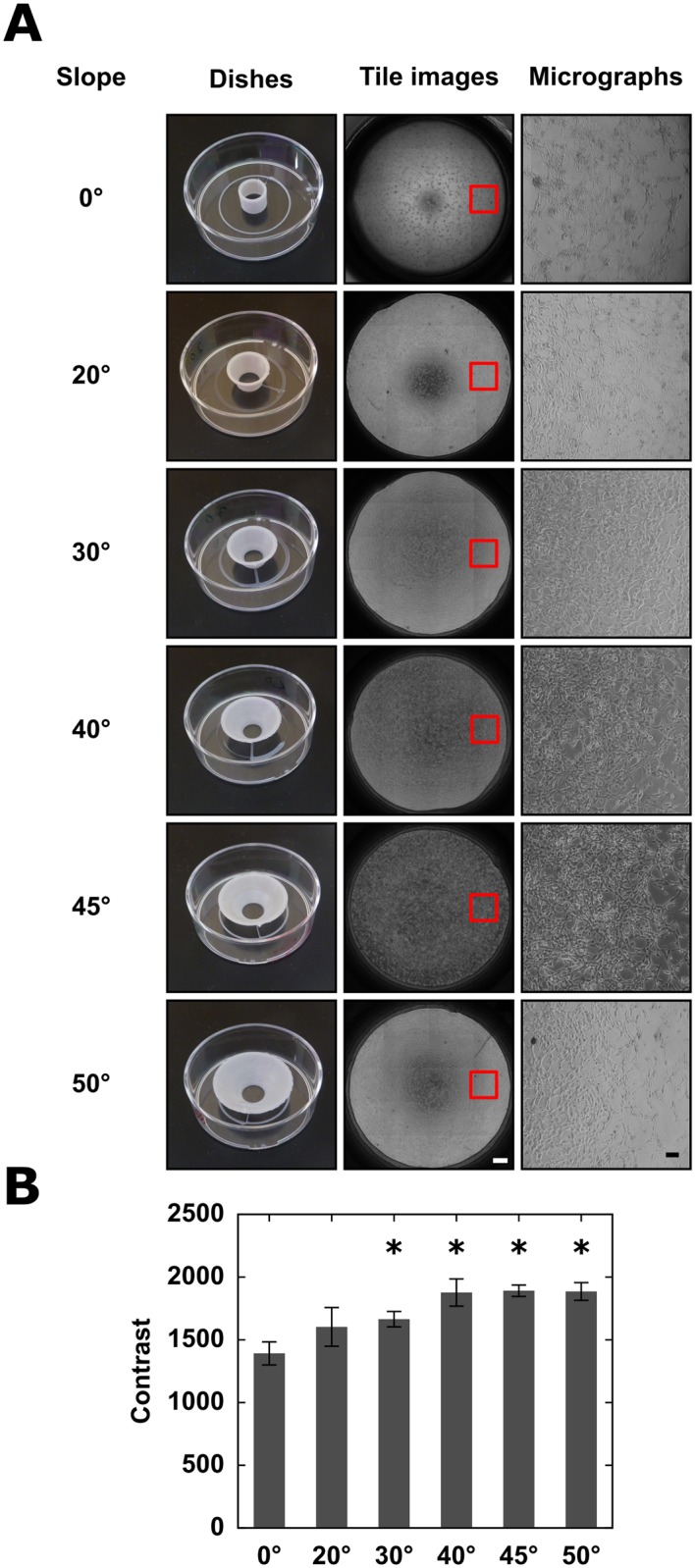
Optimization of conical wells for phase contrast optics. A: A series of wells were printed with various wall slopes or cone aperture angles (left column). 3T3 cells within the wells were imaged using an automated phase contrast microscope. The tiled mosaic fields are shown in the middle column (scale bar: 500 *μ*m). Red rectangles mark individual micrographs, that are shown in the right column (scale bar: 100 *μ*m). The best optical image corresponds to a DMEM-PLA contact angle of 40°. B: Quantitative analysis of image contrast. For each well we assigned a contrast quality value, and data is pooled from *n* = 3 parallel sets of wells. Asterisks denote significant (*p* < 0.05) difference from the image obtained in cylindrical wells, error bars indicate standard deviation.

PLA wells can be shaped in such a way that minimizes the deterioration of phase contrast optics by eliminating the curvature of the medium interface. If wells have an appropriate conical shape, the equilibrium contact angle between the wall and the medium is achieved by a flat medium-air interface. To determine the suitable slope angle of the conical surface, we made a series of models with angles ranging from vertical to outward leaning cones with an aperture angle of 100°. As Fig 3 demonstrates, for DMEM within a PLA container the phase contrast quality gradually improves until the aperture of the cone reaches 80°, i.e. the slope of the walls is 40 °. This improvement reflects both a decreasing curvature of the medium surface and an increasing distance of the medium-wall contact area.

#### 3.2.3 PLA structures in tissue culture dishes

We demonstrate the utility of the approach by some simple 3D printed structures in 35 mm culture dishes that are useful in various experimental studies. Such dishes are convenient for time-lapse studies as they offer better phase contrast images than 24 or 96 well plates.

**Fig 4:**
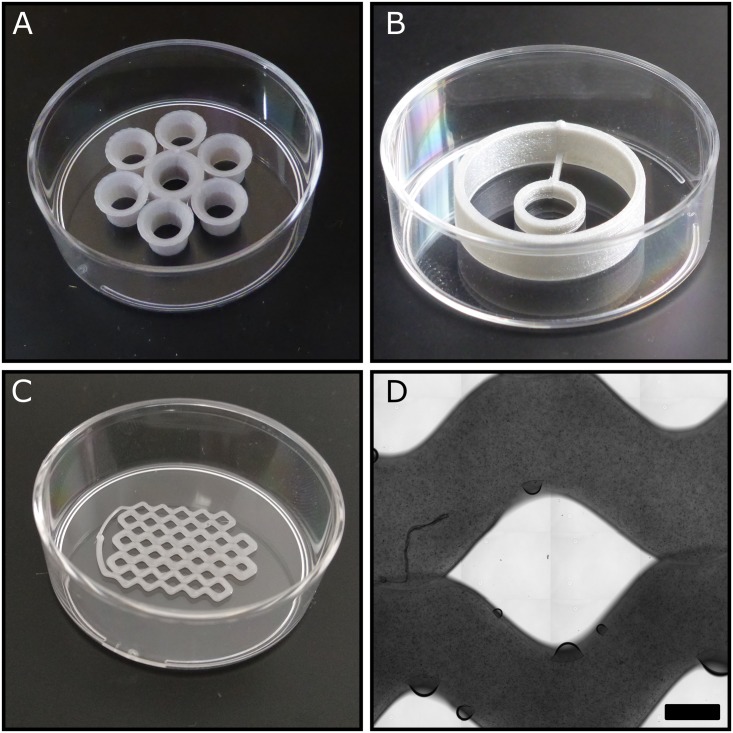
Various 3D printed culture dish configurations. A: Seven-well dish with a sloped wall to provide optimal optical quality. B: A double ring dish to control cell spreading and medium volume. C: A grid with 38 rectangular cells to store manually sorted cells. D: Phase contrast micrograph of a single PLA layer adhering to a tissue culture substrate, part of the cell sorter grid shown in panel C. Air bubbles indicate small areas with imperfect contact between the PLA layer and the underlying substrate. Scale bar: 500 *μ*m.

Dish 1 (Fig 4A) allows to maintain seven independent cultures within the same 35 mm dish, without sharing media and without deterioration of optical quality. Each well consists of a 3 mm tall cylindrical wall, which expands into a conical surface of 1 mm high with the previously determined apex angle of 100°. Dish 2 (Fig 4B) contains a small ring to limit the spreading of cells and a higher, larger diameter outer wall to set the volume of medium within the same 35 mm dish. Limiting cell spreading to a certain area can be used to locally set cell density. In particular, high cell densities can be maintained with a high medium volume/cell ratio, enabling long term observation of cultures [18]. Dish 3 (Fig 4C and 4D) contains 1 mm^2^ mini-wells that can be used to isolate cell clones, possibly obtained with a single cell sorter [19]. A microscopic image of the single layer PLA wall indicates the resolution limits obtainable with such technology: the 0.4 mm wide extrusion nozzle yields 120 *μ*m tall and 920±90 *μ*m wide PLA barriers on the tissue culture plastic. The mean area between the barriers was determined to be 1.82 mm^2^, while the standard deviation of individual cell areas was found to be 1.6.

#### 3.2.4 Biocompatibility of PLA structures

**Fig 5:**
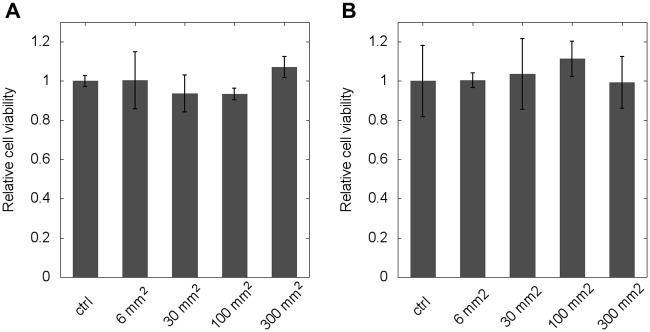
Cytotoxity assay for PLA exposure. Cells were grown in the presence of PLA surfaces for two days after which their protein content was determined by an SRB assay. Data are shown as average of *n* = 4 parallel experiments for two cell ines (A: 3T3 cells, B: p31 cells) and the effect of treatment is expressed relative to untreated controls. Error bars represent standard deviation.

To test the acute (short-term) cytotoxicity of fusion-deposited PLA structures, we exposed two well-established cell lines, 3T3 fibroblasts and p31 mesothelioma tumor cells, to various amounts of PLA. After two days in culture, the total protein content of the cells, as a viability score, was determined by the SRB assay. As Fig 5 indicates, the viability of the cells was not affected by the presence of PLA.

**Fig 6:**
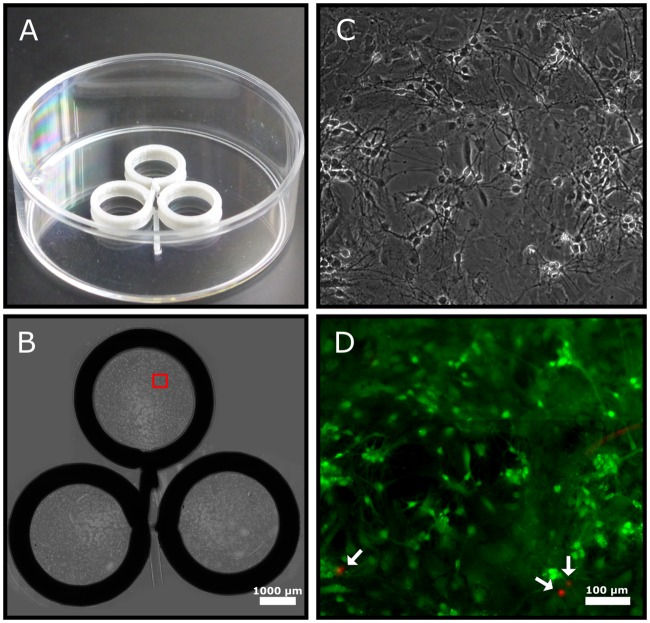
Long-term biocompatibility assay with primary hippocampal neuron culture. A: A triple ring dish used for cell culture. B: To achieve high cell density, cell spreading is restricted within the rings (diameter: 6 mm, height: 1.8 mm). A tiled image of phase contrast micrographs demonstrate the optical quality of the setup. Scalebar: 1000 *μ*m. The red rectangle marks a region shown in higher magnification in panel C. D: Composite fluorescent image to evaluate cell death within the same region shown in panel C. Live cells are labelled by calcein (green), while dead cells are labelled by ethidium (red) and marked by arrows.

Long term cytotoxicity was assayed using a primary hippocampal neuron culture (Fig 6). Cells were seeded onto Poly-L-lysine-coated surfaces at a density of 900 cells/mm2. The culture dish contained three PLA rings to limit cell spreading and to maintain high medium/cell ratio. These conditions are favorable for long-term cell culture. After 7 days in vitro, cell viability was determined using a fluorescent live/dead staining kit, which indicated a cell death rate of less than 2.2.

## 4 Conclusions

3D printing is becoming wide-spread in life sciences, either to provide cost-effective alternative to existing experimental devices, or to fabricate highly specialized structures that are specifically designed to meet certain experimental requirements. To effectively transfer 3D printing know-how, some uniformization—a common language—is required. Our approach can represent a variety of objects by sufficient detail and precision, yet in a conceptionally clearer form than a sequence of machine-specific low-level movement commands. We hope that this platform will thus facilitate both the distribution as well as the development of new, experiment-specific devices or in vitro cell technologies.

## Supporting information

S1 Appendix Example codes using the gCodeAPI. A cylinder surface is defined in C# (A) or python (B) language.
(PDF)
