# Supplementary material for: Software tools for cell culture-related 3D printed structures
Source: PLoS One. 2018 Sep 4;13(9):e0203203. doi: 10.1371/journal.pone.0203203 (PMC6122815; doi:10.1371/journal.pone.0203203)
Supplement: S1 Appendix — A cylinder surface is defined in C# (A) or python (B) language. (PDF) [file pone.0203203.s001.pdf]

S1 Appendix. Example codes using the gCodeAPI. A cylinder surface is defined in C# (A) or python (B) language.

A

C# code example

```
using GCodeAPI;

namespace PetriTaskFileTutorial
{
    class Program
    {
        static void Main(string[] args)
        {
            var task = new PetriTask("R1-D6 ", "Single ring (Diameter: 6 mm; Height: 1.8 mm)");

            double radius = 3.0d;   // radius of circle
            double stepZ = 0.2d;    // distance between two layers
            int maxZ = 9;           // number of layers
            double zOff = 0d;       // current Z offset.
            GCodeCollector code = task.Code;
            GCodeSpecial.ExtrudeRatio = 0.0405d;
            code.addCode(new GCodeLine(x: -radius, y: 0, speed: 9000d, mode: GCodeLine.SpeedModes.Fast));
            code.addCode(new GCodeLine(z: 0d, speed: 9000d, mode: GCodeLine.SpeedModes.Fast));

            for (int z = 0; z < maxZ; z++)
            {
                code.addCode(GCodeCircle.getCircleByRadius(
                        speed: 500,
                        axisAngle: 0,
                        radius: radius,
                        extrude: GCodeSpecial.ExtrudeCircle(radius)));
                zOff += stepZ;
                code.addCode(new GCodeLine(z: zOff, speed: 9000d, mode: GCodeLine.SpeedModes.Slow));
            }

            task.Save(@"C:\Users\Documents\PetriPrinter\R1D6.ptf");
        }
    }
}
```

B

python code example

```
import sys
import clr
clr.AddReferenceToFileAndPath ('gCodeAPI.dll')
from GCodeAPI import *

def Name():
    return "R1-D6"

def Description():
    return "Single ring (Diameter: 6 mm; Height: 1.8 mm)"

def Code():
    radius = 3.0    # radius of circle
    stepZ = 0.2     # distance between two layers
    maxZ = 9        # number of layers
    zOff = 0        # current Z offset.
    code = GCodeCollector()
    GCodeSpecial.ExtrudeRatio = 0.0405
    code.addCode(GCodeLine(x = -radius, y = 0, speed = 1200, mode = GCodeLine.SpeedModes.Fast));
    code.addCode(GCodeLine(z = 0, speed = 1200, mode = GCodeLine.SpeedModes.Fast));

    for x in range(0,maxZ):
        code.addCode(GCodeCircle.getCircleByRadius(speed = 500, axisAngle = 0, radius = radius,
        extrude = GCodeSpecial.ExtrudeCircle(radius)))
        zOff += stepZ
        code.addCode(GCodeLine(z = zOff, speed = 1200, mode = GCodeLine.SpeedModes.Slow))
    return code;
```
